# Obstetrical Pocket Phantom

**Published:** 1896-07

**Authors:** 


Obstetrical Pocket Phantom. By Dr. K. Shibata, Specialist in Gynecology and Obstetrics, Tokio, Japan ; Physician to the Woman's Clinic at the University of Munich. Preface by Prof. Franz von Winckel. With eight illustrations, one pelvis and two jointed manikins. Translated from the third edition by Ada Howard-Andenried, M. D., Physician to the Children's Clinic at the Woman's Hospital, Philadelphia. Price, $1.00. Philadelphia: P. Blakiston, Son & Co., 1012 Walnut street. 1895.

This booklet of twenty pages gives in the text all the presentations and positions in concise form. There is an accompanying pasteboard pelvis and two jointed pasteboard phantoms, one with the fetus received from the side, the other from the front.

They will prove very useful to students, midwives and nurses in demonstrating the mechanism of labor where a manikin and fetus would not be obtainable, and might perhaps be of service in the pocket of the every-day practitioner. In the preface of the first edition Prof. F. von Winkel, of Munich, says it "may serve as a convenient and inexpensive substitute for the pelvis and manikin." E. C.

